# Supplementary material for: Are carbon emissions trading and green financial instruments synergistic? -Comprehensive quantitative research based on content analysis
Source: PLoS One. 2024 Mar 7;19(3):e0298601. doi: 10.1371/journal.pone.0298601 (PMC10919601; doi:10.1371/journal.pone.0298601)
Supplement: S1 Data — (ZIP) [file pone.0298601.s001.zip › Data/地级市Z_F工作报告.zip!/╣π╬≈/╬α╓▌╩╨/└┤╫╘╣1⁄2╓┌║┼í╛┬φ┐╦╩2╛▌═°í┐_2006.docx]

各位代表：

现在，我代表市第十一届人民政府向大会作工作报告，请予审议，并请市政协各位委员和其他列席人员提出意见。

过去六年工作的回顾

市十一届人民代表大会第一次会议以来，本届政府在自治区党委、政府及市委的领导下，在市人大及其常委会的监督和市政协的支持下，坚持以邓小平理论和“三个代表”重要思想为指导，全面落实科学发展观，紧紧依靠全市人民，抢抓机遇，开拓进取，克服“非典”、禽流感等突发公共事件带来的不利影响，战胜了特大洪灾、历史罕见的地质灾害，全力推进工业化、城镇化和农业产业化，圆满完成了市十一届人民代表大会历次会议确定的各项目标任务。

过去的六年，是全市综合实力提升较快，人民群众得到实惠较多，经济社会持续快速健康发展的六年；是加快城镇化建设进程，人居环境显著改善，城乡面貌发生巨大变化的六年；是全面加强物质文明、政治文明、精神文明建设，构建和谐梧州取得显著成绩的六年。

一、国民经济持续快速健康发展，综合实力显著增强

六年来，全市国民经济发展呈现速度加快、总量翻番、效益提高、后劲增强的良好局面。地区生产总值由2000年的127.08亿元增加到2005年的228.4亿元，年均增长10.8%，按2005年常住人口计算，人均地区生产总值超过1000美元。财政收入由2000年的9.88亿元增加到2005年的20.25亿元，年均增长15.43%。2005年，全市规模以上工业总产值、规模以上工业增加值、工业企业实现利润、全社会固定资产投资等指标对比2000年实现翻番。全市经济结构明显优化，三次产业比例由2000年的32.9∶30.3∶36.8转变为2005年的21.8∶42.7∶35.5。今年前三季度，各项主要经济指标目标任务如期实现，国民经济继续保持快速发展态势。

农业和农村经济稳步发展。全力实施农业五大措施，农业结构调整迈出坚实步伐，优势特色产业发展取得新突破。全市农业增加值由2000年的41.8亿元增加到2005年的49.8亿元，年均增长4.9%。农业优势产品基地化生产初步形成，一批涉农企业发展壮大，有7家农业企业被评为自治区级农业产业化重点龙头企业。农业产业化经营初具规模，12个农产品生产基地和9个养殖基地通过自治区无公害标准化生产认证，主要农产品生产能力和农产品质量安全水平明显提高。县域经济发展迅速，2001年至2005年，县域生产总值年均增长12.5%。社会主义新农村建设试点工作全面启动，27个新农村建设试点取得良好成效。农业基础设施建设全面加强，农业科研和技术推广步伐加快，农村其他工作均取得新发展。

工业经济快速增长。工业增加值由2000年的33.3亿元增加到2005年的81亿元，年均增长16.7%。工业对经济增长的贡献率由2000年的34.3%提高到2005年的49.5%。规模以上工业企业盈亏相抵后实现利润由2000年的0.1亿元增加到2005年的3.95亿元，年均增长106.8%。国有企业产权制度改革基本完成，走在全区前列。国有资产的监管进一步规范。集体企业改革工作稳步推进。支柱产业迅速成长，优势企业数量增加，初步形成了林产林化、日用化工、食品、制药、宝石加工等具有地方特色的五大支柱产业。市工业园区建设取得较好成绩，完成固定资产投资9.5亿元，其中基础设施完成投资3.6亿元，实现工业总产值4亿多元。全市工业化率由2000年的0.8提高到2005年的1.63，进入工业化初级阶段。

第三产业保持强劲增长态势。第三产业增加值由2000年的46.8亿元增加到2005年的81亿元，年均增长9.4%。社会消费品零售总额年均增长11.8%。服务业结构进一步优化，市场培育力度进一步加大，商贸设施日趋完善，功能更加齐全。初步形成了以宝石城、两广批发市场、竹湾农副产品批发市场等为龙头，辐射桂东南和粤西地区的大市场、大流通格局。交通运输业取得长足发展。旅游业实现新突破，累计接待游客990.75万人次，实现旅游总收入43.16亿元，荣获“中国优秀旅游城市”称号。

二、招商引资实现新突破，对外开放水平不断提高

招商引资取得新成效。主动融入珠三角经济圈，积极参与多区域经济合作，创新招商方式，加大对重点区域、重点产业、重点企业的招商引资力度，并取得显著成效。以宝石为媒，成功举办了三届梧州国际宝石节，提高了我市知名度，扩大了在国内外的影响力，有力地促进了招商引资工作。全市累计引进外来投资项目1263个，其中境外项目274个，实际利用外资4.15亿美元，年均增长16.9%；境内合作项目989个，实际到位资金99.5亿元，年均增长51.9%。外来投资项目中东部产业转移项目584个，占总数的46.2%；到位资金61.3亿元，占外来投资总量的46.1%。2005年，引进工业项目284个，占全市引进项目总数的64%；工业项目到位资金46.3亿元，占全市到位资金的69.8%；全市三资企业实现工业总产值44.84亿元，年均增长39.5%。

对外经济贸易持续快速发展。全市外贸进出口总额累计11.91亿美元，年均增长31.2%。外贸出口持续高位增长，其中，2005年外贸出口总额2.46亿美元，比2000年增长2.2倍。对外交流与合作取得新成效，与130多个国家和地区建立了经贸关系，与东盟各国和港澳台的经贸合作进一步加强。多次荣获全区实际利用外资和外贸出口先进单位称号。

三、城市建设力度加强，市容市貌变化巨大

城市基础设施建设成效明显。加大城市建设力度，以开展三百项目大会战为契机，全面推进城市面貌“三年大变”建设。仅2003年到2005年，城市基础设施建设完成投资达12亿元，完成城市道路改造41.4公里，改造桥梁5座，完成了70多条道路的绿化美化和综合管线改造工程，新增排水渠25公里，完成城市主干道沿街建筑立面改造33万平方米，新安装各种路灯6万多盏，城市交通、能源、水电、通信等基础设施日臻完善。这三年，是城市建设投入最大、发展最快、成绩最为显著的三年。

市容环境呈现新面貌。积极推进旧城区改造，建设新城区，打造城建精品，一批标志性建筑相继建成。拓宽改造后的西堤路，成为城市交通的主干道和休闲观光带；新建成的鸳江丽港，成为别具特色的旅游购物中心；改造后的骑楼城，蕴含丰富岭南文化内涵，成为一道亮丽的旅游风景线。城市美化、绿化、亮化程度提高，城市品位得到了提升，荣获自治区第五届市容环境综合整治“南珠杯”竞赛A类城市特等奖。全市城镇化进程进一步加快，2005年，城镇化水平达32.1%，与全区平均水平基本持平。

四、重大项目建设加快，投资拉动经济增长成效明显

重大项目建设取得突破性进展，带动经济发展效果突出。“十五”规划提出的五大工程全部实现预期目标，全市人民盼望多年的长洲水利枢纽、洛湛铁路梧州段、南梧高速公路岑梧段、桂梧高速公路马江至梧州段等重大项目顺利推进，河东防洪堤和桂梧高速公路苍郁段已经建成并投入使用，经济社会加快发展的基础进一步夯实。“十五”累计完成全社会固定资产投资261亿元，是“九五”的2.75倍；新开工投资亿元以上项目56个，是“九五”的4.3倍。投资对经济增长的贡献率年均超过60%。

五、防灾减灾能力增强，人民生命财产安全得到保障

加强自然灾害、事故灾害、公共卫生和公共安全等监测预警体系建设，制定和完善各种公共突发事件防控预案，特别是防洪预案的制定实施，使我市防洪抗灾应急工作步入规范化轨道。建立健全信息报告、应急救援、社会动员机制，应对处置公共安全突发事件和抗灾减灾能力不断增强，夺取了抗击“非典”和防控高致病性禽流感的重大胜利，战胜了2005年“6·23”百年一遇的特大洪灾和2006年“6·8”历史罕见的地质灾害，最大限度地降低了人民群众生命财产损失。2000年以来，完成了24421户因灾倒塌民房恢复重建，妥善安置了受灾群众的生产生活。地质灾害整治加快推进，水上居民搬迁顺利开展。目前，地质灾害治理已被列入国家项目，共完成地质灾害危房搬迁783户，水上居民搬迁199户。

六、财政收入和城乡居民收入增加，人民生活水平稳步提高

财源建设成绩突出。2005年，全市纳税10万元以上企业达894家，比2000年增长1倍，纳税总额占全市财政收入的49.1%。2005年，全市财政收入首次突破20亿元，比2000年增长1倍。

城乡居民收入明显增加。2000年到2005年，全市农民人均纯收入由1682元提高到2575元，年均增长8.9%；城镇居民人均可支配收入由4839元提高到8118元，年均增长10.9%。

社会保障和就业工作成效显著。城镇职工基本养老保险、失业保险、医疗保险、工伤保险、生育保险和城镇最低生活保障稳步推进，社会保障体系初步建立。城镇登记失业率控制在4.5%以内，就业和再就业工作进入全国先进行列。

人居环境得到较大改善。市区绿化覆盖率达45.61%，建成区绿地率达43.97%，人均公共绿地7.76平方米，3项指标已接近国家园林城市标准，排在全区前列。6种主要污染物排放总量均控制在自治区下达的指标以内。市区空气质量优良率和市区集中饮用水源水质达标率均达到100%。

七、社会事业全面进步，精神文明建设不断加强

科技创新取得新成就。全市取得自治区级科技成果96项，市级科技成果119项。承担国家“十五”重大科技项目通过国家验收。科技创新支撑经济综合指标、科技活动产出水平、科技环境改善指标全面提高。科技特派员试点工作取得新成效。

教育发展再上新台阶。教育投入不断加大，改造农村中小学危房27.4万平方米。“十五”全市累计投入教育经费34.8亿元，其中财政性投入23.57亿元，完成教育基础设施建设投资7.81亿元，分别比“九五”增长115.04%、112.59%和103.14%。“普九”攻坚和“两基”巩固提高工作取得新突破，各级各类教育事业改革取得新进展。广西大学梧州分校申本成功，梧州学院正式成立。

文化体育卫生事业蓬勃发展。群众文化活动广泛开展，公共文化服务体系初步形成。广播电视“村村通”工程顺利实施，在全国率先实现市区和县城有线电视数字化整体转换，梧州电台与广东电台联合发展取得丰硕成果。群众体育活动活跃，竞技体育成绩显著，我市运动员在国内外重大比赛中获金牌15枚、银牌5枚、铜牌10枚。卫生事业健康发展，城乡医疗卫生服务体系、全民公共卫生体系和突发公共卫生事件应急机制基本建立，三级医疗预防保健网络逐步完善。新型农村合作医疗试点工作取得新成效。

精神文明建设成效显著。全市文明社区、文明村镇、文明单位、文明行业、文明城市等各项创建活动全面推进，连续被评为第三、第四轮自治区文明城市，13个单位获全国文明单位、文明村镇、精神文明建设先进单位称号，22个单位被评为自治区文明集体。《公民道德建设实施纲要》得到深入贯彻落实，社会主义荣辱观教育深入人心，未成年人思想道德建设工作进一步加强。国防动员和民兵预备役建设成效显著。军警民共建活动深入开展，再次荣获“全国双拥模范城”称号。

建设平安梧州活动全面开展。依法严厉打击严重刑事犯罪活动，各种刑事犯罪得到有效遏制。社会治安综合治理进一步加强，治安防控体系逐步完善。重视群众信访工作，全面开展“大接访、大调处”活动，群众反映的突出问题得到妥善处理，集体上访、越级上访和群体性事件明显减少。法律援助网络和制度建设进一步完善，人民调解工作效果明显。建立健全安全生产监管体制，加强安全生产设施建设，有效遏制重特大事故发生，为经济社会快速发展创造了良好环境。

其他各项事业稳步推进。各民主党派工作条件得到改善。人口和计划生育工作取得明显进步。资源合理开发利用，环境保护和生态建设得到加强。工会、妇女儿童、青少年、老龄、残疾人等各项事业稳步发展，人事、民政、扶贫、机构编制、审计、统计、外事、侨务、档案、修志、新闻出版、地震、人防、社会科学、宗教等各项工作取得了新的成绩。国家安全、金融保险、工商、税务、气象、水文、电信、邮政、供电、烟草、质量技术监督、海关、海事、食品药品监督、检验检疫等中直、区直驻梧单位为我市经济社会发展提供了有力保障，作出了积极贡献。驻梧部队为我市经济社会发展特别是在抗洪救灾、突发事件处置等方面发挥了重要作用。

八、政府自身建设不断加强，执政能力明显提高

自觉接受人大监督和政协民主监督，广泛听取社会各方面的意见和建议，人大代表议案、建议，政协委员提案和社会各界来信来访的办理水平进一步提高。六年来，市本级共办理市人大代表议案、建议393件，市政协委员提案1014件，按期办复率达100%。认真实施《依法行政实施纲要》。加快政府职能转变，行政管理体制改革进一步深化，积极推进政企分开、政务公开和电子政务，政府经济调节、市场监管、社会管理和公共服务职能进一步增强。政府机关依法行政水平和工作效率不断提高，行政审批制度改革不断深化。解放思想再讨论、政务环境评议、保持共产党员先进性教育和“两整一会战”等活动有效开展，公务员队伍建设进一步加强。坚持从严治政，加强政风行风建设，深入开展反腐败斗争，切实纠正部门和行业不正之风，严肃查处了一批违纪违法案件，解决了群众反映比较强烈的问题。

各位代表，回顾过去六年的工作，我们所取得的成绩来之不易，这是自治区党委、政府和市委正确领导的结果，是全市上下真抓实干、艰苦奋斗的结果，是历届市委、政府打下良好基础的结果。在此，我代表市十一届人民政府，向全市广大干部群众，驻梧部队官兵，各民主党派、工商联、人民团体以及所有关心支持我市改革开放和现代化建设事业的各界朋友，致以崇高的敬意和衷心的感谢！

过去六年的探索和实践，使我们进一步深化了对经济社会建设规律的认识，积累了许多宝贵经验。我们主要有以下6个方面深刻体会：一是必须坚持用邓小平理论、“三个代表”重要思想和科学发展观统领经济社会发展全局，始终坚持以经济建设为中心，以加快发展为主题。二是必须坚持改革开放，充分发挥自身优势，努力探索具有梧州特色的经济发展新路子。三是必须坚持与时俱进，抢抓机遇，积极参与多区域经济合作，加强投资和项目建设，不断为经济和社会发展注入活力。四是必须坚持以人为本，执政为民，始终维护好广大人民群众的切身利益。五是必须坚持物质文明、政治文明和精神文明同时抓，不断为改革和发展提供强大的精神动力和物质保障。六是必须坚持加强政府自身建设和公务员队伍建设，努力建设廉洁、勤政、务实、高效的法治政府。

回顾六年来的工作，我们为全市经济社会快速发展而倍受鼓舞，同时也清醒地认识到，我市经济社会发展还存在不少困难和问题：一是经济总量不大，质量不高，企业竞争力不强，缺少大工业项目的支撑。二是财政收入总量偏小，财政收支矛盾突出。三是县域经济发展还有差距，农业基础薄弱，农业产业化水平较低。四是社会保障体系仍待完善，就业和再就业压力较大，低收入群体生活水平还比较低。五是工业化、城镇化水平还较低。六是政府工作还存在不少薄弱环节，行政效能有待提高，机关作风、投资环境有待进一步改善。此外，我们还面临着市场、资源、技术、人才的激烈竞争和沿海及周边地区加快发展的严峻挑战。所有这些困难和问题，我们将高度重视，并在今后的工作中采取切实有效措施，认真研究解决。

今后五年工作的建议

今后五年是我市深入实施“十一五”规划，全面建设小康社会，实现经济社会更快更好发展的关键时期。根据党的十六大、十六届六中全会精神和市第十一次党代会描绘的美好蓝图和部署，政府工作要坚持以邓小平理论和“三个代表”重要思想为指导，全面落实科学发展观，以加快发展为主题，积极参与多区域经济合作，加大项目和基础设施建设力度，加快推进工业化、城镇化和农业产业化，做大经济总量，提高人民群众生活水平，改善人居环境，推动各项社会事业全面发展，努力建设富裕和谐新梧州。

根据这一要求，今后五年我们的主要奋斗目标建议安排为：到2010年，全市地区生产总值年均增长13%以上，力争比2005年翻一番；财政收入年均增长15%以上，比2005年翻一番；全社会固定资产投资累计完成600亿元以上，比“十五”翻一番；社会消费品零售总额年均增长12%以上；城镇居民人均可支配收入年均增长7%以上，农民人均纯收入年均增长8%以上；人口自然增长率控制在11‰以内；城镇登记失业率控制在4.6%以内。

一、着力推进新型工业化，进一步壮大经济总量

全面实施“工业强市”战略，按照自治区“一轴两廊”工业发展新格局的要求，以壮大工业经济总量为中心，充分发挥我市资源优势，坚持新兴工业与传统工业并重，走新型工业化道路，加快推进工业化进程。到2010年，力争全市规模以上工业总产值达到400亿元，规模以上工业增加值超过150亿元。

积极承接东部产业转移。充分发挥我市毗邻粤港澳的区位优势和资源优势，主动融入泛珠三角经济圈，改造传统产业，发展新兴产业，推进产业优化升级。积极引进集约用地、投资强度大、污染较低、科技含量高的项目，延长产业链，形成产业集群，全力打造承接东部产业转移的外向型工业基地。

做大做强支柱产业和优势龙头企业。在原有的林产林化、日用化工、制药、食品和人工宝石等五大支柱产业的基础上，把冶金、电力、建材、机械（造船）和纺织等产业培育成为新的支柱产业。坚持扶优扶强，在政策上、资金上重点扶持一批优势龙头企业。到2010年，力争培育形成年销售收入超50亿元的企业1家以上，超30亿元的企业3家以上，超10亿元的企业5家以上，超5亿元的企业10家以上，超亿元的企业30家以上。在此基础上，形成年销售收入超100亿元的产业1至2个。以此带动支柱产业的突破性发展，把我市建设成为桂东工业基地。

努力培育产业集群。坚持把内涵扩大再生产、延长产业链、发展产业集群作为发展工业经济的重大举措，以支柱产业、优势龙头企业为核心，打造各具特色的产业集群。市区工业重点发展林产、医药、食品、机械（造船）、日用轻工、纺织、化工、电力以及宝石加工等产业集群。岑溪市发展以石材为主导的建材、有色金属冶炼为主导的有色金属产业集群。苍梧县发展以松脂及深加工为主导的林化、有色金属冶炼及压延业为主导的冶金产业集群。藤县发展以钛白粉为主导的化工、林浆纸为主导的林产产业集群。蒙山县发展以蚕丝为主导的纺织、水泥为主导的建材和木薯淀粉为主导的食品产业集群。

全力推进重大工业项目建设。举全市之力，抓好林浆纸一体化项目、年产30万吨木薯燃料乙醇项目、年产120万吨新型旋窑水泥项目、4×60万千瓦火电项目、桂东核电项目等一批重大项目的建设或前期工作。继续抓好三威年产30万立方米均质刨花板、林脂一体化、制药基地、蛋白肠衣扩建、长洲水利枢纽、旺村水利枢纽、纺织工业基地、年产15万吨氯碱、金属铟、梦都美地毯等一批重点项目的建设。扩建和新建一批110千伏、220千伏和500千伏的输变电工程。力促这批项目早日开工并尽快投产，培育新的经济增长点。

大力发展园区经济。把工业园区和工业集中区建设放到重要位置，坚持“一园多基地”的模式，提高项目承载能力，通过合作投资、联手开发、利益共享等办法，扩大规模，增强实力，形成区域经济新高地。积极落实土地政策，努力提高用地效率，确保工业建设用地的供应。加大存量土地盘活力度，清理部分项目用地，把有限的土地用在急需的新项目上。采取市场化运作方式，鼓励有实力的投资者参与工业集中区开发、建设和经营，形成以工业园区为龙头，龙圩、塘源和赤水临港工业基地为支撑的工业建设格局。积极开拓南梧、桂梧、苍郁高速公路以及市区至县城交通干线为轴线的通道工业经济带，形成一批产业明确、分工协调、资源互补、利益共享的各具特色的工业基地。到2010年，力争市工业园区建成区面积达到5平方公里，固定资产投资累计达25亿元，实现工业总产值60亿元以上。

努力打造现有工业品牌。鼓励企业创名牌产品，抓好骨干企业的自主创新，培育发展创新型中小企业。发挥财政的引导和支持作用，加快发展具有自主创新能力、拥有自主知识产权的“两自型”企业。支持企业技术进步，继续实施一批技术创新和技术改造项目，应用高新技术和先进技术改造提升传统产业。到2010年，力争全市名优产品销售收入占全市规模以上工业企业销售收入的比重达到50%以上。

二、着力推进农业优势产业发展，扎实开展社会主义新农村建设

今后五年，要着力发展农业优势产业，推进农业产业化进程，千方百计增加农民收入，改善农村生产生活条件，全面推进社会主义新农村建设。

继续调整农业产业结构，大力发展优势特色产业。在全面贯彻国家粮食生产政策、确保粮食安全的基础上，着力推进亚热带名优水果、木薯深加工、桑蚕与茧丝绸一体化、肉桂八角香料、速丰林与林浆纸一体化、三黄鸡、瘦肉型猪、江河网箱养殖、六堡茶、中草药等十大农业优势特色产品和产业开发。大力培育、扶持、引进和壮大一批农业龙头企业，延长农业产业链，促进农产品加工增值，形成“壮大一批龙头企业、培育一批特色产业、建成一批生产基地、带动一方农民致富”的农业产业化发展格局，构建农民增收的长效机制。

加强基础设施建设，发展农村社会事业。继续抓好农田水利建设，重点抓好病险水库除险加固和中小型灌区节水改造，努力增加有效灌溉面积，改善农业生产条件。进一步抓好农村饮水工程建设，着力解决群众饮水困难和饮水安全问题。继续开展乡村公路建设大会战。进一步完善农村供电网络，提高农村安全用电质量。大力发展以沼气为主的农村生态能源建设，改善农村生态环境和村容村貌。加大农村教育、文化、卫生等基础设施投入，加快发展农村社会事业。

加大新品种新技术推广力度，提高农业科技贡献率。大力推广农业优良品种和先进适用技术，加快农业科技成果应用转化。加快无公害标准化农产品生产基地建设，确保主要优势农产品生产基地通过自治区级无公害标准化生产认证，提高农产品竞争力。进一步健全农产品质量安全监测体系，提高农产品质量安全水平。加大农机应用推广力度，积极调整农机装备结构，努力提高农业机械化综合作业水平。进一步做好各种重大动植物疫病的防控工作。继续实施“阳光工程”，加强对农民的综合培训，提高农民素质，培养新一代有文化、懂技术、会经营的新型农民。继续抓好农村劳动力转移就业工作，把农村劳动力转移就业作为增加农民收入的一项新兴产业来抓，确保取得成效。

加强市场体系建设，搞活农产品流通。加大政府对农业流通领域的扶持力度，依托特色农产品建设专业农产品批发市场，不断完善农业信息进村入户工程，继续发挥鲜活农产品“绿色通道”的作用，构建稳定的农产品营销网络。

深化农村综合改革，加大发展扶持力度。坚持“多予少取放活”的方针，落实各项惠农支农政策。强化涉农收费和农资价格监管。做好乡镇综合配套改革工作，健全和强化基层农业技术服务体系，确保中央和自治区各项涉农方针政策落到实处。

大力发展县乡工业，做大做强县域经济。尤其要抓好资源开发加工型工业、配套工业和劳动密集型工业的发展，通过壮大工业总量来增强工业反哺农业的能力，形成以城带乡、相互促进、协调发展的格局。力争全市有1至2个县（市、区）进入广西经济发展十佳县（市、区）行列。

三、着力推进多区域经济合作，进一步提高对外开放水平

参与多区域经济合作，既是我市经济社会发展的必然要求，也是提速发展的正确选择。为此，我们必须抢抓机遇，进一步发挥区位优势，把融入珠三角经济圈作为主导，东融西联，主动参与多区域经济合作。

努力优化环境，主动融入珠三角。进一步转变观念，拓宽思路，学习珠三角地区敢为人先、搁置争议、抢抓机遇的精神，借鉴珠三角地区发展经济的思路、对策和经验，积极进行制度创新和机制创新，特别是要在管理方式和运作模式上主动与珠三角地区接轨。大力改善服务环境，提高办事效率，简化审批程序，进一步规范中介机构服务性收费，鼓励金融机构对重点项目和有潜力的项目给予资金支持，为外商提供一流的服务，增强招商竞争力。

加快对接步伐，自觉接受东部经济辐射。根据珠三角地区的发展方向，结合我市的发展现状，充分利用我市资源和产业优势，抓住珠三角资金、技术和市场转移的机遇，加快推进与珠三角的产业对接、市场对接，实现区域内资源有效利用和互利共享，大力引进资源能源开发型项目，带动工业产品的深度加工、深度开发和资源的综合利用。

发挥区位优势，加强与东盟和大西南地区的经济合作。紧紧抓住自治区实施“一轴两翼”区域经济合作战略的机遇，充分利用我市面向粤港澳、背靠大西南的良好区位，加快基础设施建设，强化我市作为大西南地区东出海通道的地位和作用，不断拓展与东盟国家和大西南地区的经济合作，力求在投资、贸易、资源开发利用、交通物流、旅游等领域的合作取得新突破。

创新招商引资方式，提高利用外资质量。以港澳台和珠三角地区作为招商引资的重点区域，积极拓展与长三角地区以及日韩和东盟各国的投资合作，采取政府招商、企业招商、以商招商、政府与政府合作推动产业转移等多种方式，吸引更多企业和产业进入我市。抓好资源招商和产业招商，积极引进大公司和大企业来我市设立生产基地、配套加工基地和服务业外包基地，建设一批投资规模大、就业能力强、财政贡献率高的重大工业项目。通过产业的承接，使我市真正成为与泛珠三角一体化的区域研发制造中心。

大力发展对外贸易，提高经贸合作水平。参与长三角、泛珠三角、“一轴两翼”经济圈的商贸合作，扩大商品的双向流通和贸易往来，实现流通领域合作的新突破，推动各类产品的出口。加强跨区域大型基础设施和公用设施的建设以及物流体系与信息网络的对接，简化通关手续，促进货物快速高效流动。重视国际化市场发展，以粤港澳台地区、东盟各国为桥梁和纽带，提高经济开放度。

四、着力推进中心城市建设，努力构建集约发展的城镇体系

坚持以规划为先导，发挥城市规划对城市建设的综合调控作用，按照相对集中和资源优化配置的原则，高起点、高水平做好城市规划。通过强化城市规划管理，切实做到科学有效地指导城市建设，提升城市品位。

全力打造中心城市。今后五年，城市建设将按照“拓展新城区与改造优化旧城区相结合，重点拓展城市新区”的要求，以建设区域性交通枢纽、旅游商贸物流中心为目标，努力打造富有特色的“岭南山水文化名城”。整合市区资源和空间形态，调整城市组团结构，逐步形成“一条轴线、多个组团”紧凑布局的组团式城市架构。引导苍梧县城有序发展，实行严格的规划管理。重点加快红岭新区和新兴三路、西堤路沿线建设，推进苍梧片区、高旺片、旺甫片开发，规划建设好环绕中心城区的长洲岛、钱鉴片、莲花山片、八宝塘片和塘源片等外围组团，拓展城市新区，扩张城市规模。以实施市区地质灾害综合治理、城中村改造和骑楼城改造等重大工程为重点，对旧城区进行保护性改造和开发建设。优化河东、河西、大塘片旧城区居住环境，完善城市公共设施，实现旧城换新貌的目标。到2010年，市区建成区面积达50平方公里以上，市区人口超过65万人。

加快县城和重点镇建设。把岑溪市建设成为中等城市，到2010年，建成区面积达20平方公里，市区人口达23万人，城镇化率达35%。苍梧县城要充分发挥紧靠中心城市和交通要道的优势，建设成为梧州市的物流中心和新工业基地，到2010年，建成区面积达13平方公里，城区人口达13万人，城镇化率达30%。藤县县城要抓住高速公路建设、西江航道整治、新港码头以及梧州林浆纸工业项目建设等机遇，加快向中等城市迈进的步伐，到2010年，建成区面积达15平方公里，城区人口达13万人。蒙山县要依托桂林旅游圈和良好的自然条件，发挥旅游和绿色产业优势，继续推进县城基础设施建设，扩大城区规模，到2010年，县城建成区面积达5平方公里，城区人口达5万人。完善和强化中心城区作用，辐射和带动南部、西部、北部、西北部和中部等5条城镇带的发展。构建中心城市、副中心城市、县城中心城镇、重点镇和一般集镇梯次分布、集约发展的城镇体系。

加快立体交通网络建设。全力推进南宁至梧州、桂林至梧州、广州至梧州以及岑溪至罗定、平南至梧州等高速公路建设；大力实施“通达”工程和“通畅”工程，完善区域内公路网络，不断改善县乡村道路，努力实现“县县通二级路、镇镇通油路、村村通等级路”的目标；力促洛湛铁路梧州段尽快完工，力争柳州至梧州至肇庆铁路、南宁至广州快速铁路梧州段早日开工建设；推进西江航道整治，提升梧州至贵港段、长洲水利枢纽至广东段航运能力，加快梧州港赤水圩作业区、龙圩深水港码头建设和李家庄码头扩建，充分发挥西江黄金水道的优势和作用。利用现有机场资源，尽快恢复区内外航线。努力把我市打造成为广西乃至西南地区与粤港澳联接的交通枢纽。

完善城镇功能，提高发展质量。建设完善城镇水、电、气供应系统。实施旧城区供水管网改造与扩建，配套铺设新区供水管网，全面提高城区的供水质量和能力。加强岑溪、苍梧、藤县、蒙山和其他小城镇的水源工程和供水管网建设，保障城镇供水安全。加强住宅区管理，不断完善城镇居住功能。实施居住区开发示范工程，鼓励开发建设特色小区。扩大住宅供给，加强廉租住房建设，规范经济适用房管理，改善城镇居民特别是低收入群体的居住条件，实现城镇居民人均住宅建筑面积达到20至25平方米。努力建设管理有序、服务完善、环境优美、治安良好、生活便利、人际关系和谐的新型城市社区。

大力实施“城乡清洁工程”。立足于改善城乡环境卫生形象，提升城镇档次，着力解决好城市管理中存在的“脏乱差”和违章建筑等突出问题以及老百姓关注的热点难点问题。进一步提高市民群众的环境卫生意识，引导市民群众形成良好的卫生习惯。建立健全城市管理机制，结合开展市容“南珠杯”竞赛活动，加大市容市貌特别是城郊结合部和小街小巷的综合整治力度，努力创建清洁、文明、有序的国家级园林城市。

五、着力推进商贸旅游发展，不断提高第三产业质量

加快发展服务业，是实现经济持续健康发展的重要保障。要大力发展商贸旅游，调整优化服务业结构，提高服务业发展水平。

大力发展商贸服务业。推进发展现代流通业，加快电子商贸应用，进一步改造传统商业结构和经营业态，提升商贸流通业的整体素质。加大专业市场建设培育力度，对现有批发市场进行改造和提升，建设与支柱产业、重点行业相配套的综合市场，形成一批有较强辐射力的全国性和区域性批发市场。依托高速公路、铁路站场、港口码头建设，积极推进物流园区和物流基地建设，把我市建成区域性商贸物流中心。依托宝石节，不断拓展节会产业。加快房地产业发展，加强房地产市场管理，培育房屋租赁市场，规范房地产中介服务和物业管理。培育和发展市场需求潜力大的服务行业，进一步优化服务业结构。

加快发展旅游业。抓住泛珠三角区域合作的机遇，加强与周边城市的区域旅游合作，主动融入珠三角旅游圈、大桂林旅游圈，把我市打造成为“粤港澳旅游休闲后花园”和“两广旅游集散中心城市”。加快培育和打造具有文化特色的旅游精品项目，市区重点打造一山（白云山）、一寺（四恩寺）、一庙（龙母太庙）、一塔（西江明珠塔）等景区景点，形成以白云山为主体，骑楼城、中山纪念堂、李济深故居等一批融宗教历史文化、龙母文化和现代旅游观光为一体的龙头景区。加强旅游基础设施建设，抓好蒙山太平天国封王遗址、藤县太平狮山、岑溪吉太景区群、苍梧飞龙湖休闲度假区开发。加强旅游景区景点保护，整合旅游资源，培育形成一批环绕市区龙头景区的自然资源旅游精品。以宝石、食品为载体，大力研发富有特色的旅游产品。建立旅游商品批发销售市场，规划建设土特产一条街、文化旅游商品一条街、特色美食一条街，形成具有特色的专业商业街群，繁荣梧州人气，提高旅游经济水平。

六、着力推进财税金融工作，增强对经济社会发展支持能力

加强和改善财政、金融对经济发展的引导和扶持，努力培植财源。加强财政管理，改进服务和监管，防范财政风险，确保财政金融平稳运行。

加大财源培植力度。利用财政杠杆，加大对重点税源特别是支柱税源企业的扶持力度。每年选取100家纳税大户作为重点服务对象，为他们提供优质、高效、便捷的服务，并在资金上给予扶持、政策上给予倾斜。到2010年，力争纳税100万元以上的企业达250家，并培植一批纳税超5000万元的企业，努力实现纳税超亿元工业企业的突破。强化依法治税意识，认真查处各种涉税违法行为，确保财政收入稳步快速增长。加强和规范国有资产监督管理，确保国有资产保值增值。

逐步完善公共财政体制。建立财政收入保障机制，增强财政调控能力。优化财政支出结构，在确保法定支出和重点支出的前提下，集中财力重点支持支柱产业发展和基础设施建设。扩大公共财政覆盖范围，加大对农业、科技、教育、公共卫生、计生、就业社保等事业的投入。进一步深化国库集中收付、部门预算、非税收入管理和政府采购制度改革，继续推行财政性投资评审制度和财政绩效评价制度。

加强金融环境建设。充分发挥金融促进经济发展的主渠道作用，通过构建各种融资平台，加强与金融机构的合作，拓宽融资渠道，解决资金缺乏这个制约当前经济发展的关键问题。建立健全社会诚信体系，优化金融生态环境，打造“诚信梧州”。支持金融改革和创新，加强金融监管和服务，防范和化解金融风险。推进中小企业信用担保体系建设，帮助企业增强融资能力，切实解决中小企业融资难和社会主义新农村建设资金问题。运用市场化理念，引进国内外风险投资机构到我市发展业务，实现金融与经济发展良性互动、互利共赢。

七、着力推进民生工程建设，切实让广大群众得到更多实惠

高度关注民生、提高人民生活水平是各级政府义不容辞的责任，也是构建和谐梧州的关键。必须按照构建和谐社会关于“家庭财产普遍增加，人民过上更加富足生活”的要求，从扩大就业、完善社会保障体系、理顺分配关系入手，切实为民谋利、为民造福。

切实维护群众利益。把群众关心的热点、难点问题作为政府工作的重点，突出解决好征地拆迁、拖欠农民工工资等方面的问题。加大对困难群众的帮扶力度，不断改善群众生产生活条件。推进扶贫开发，扩大智力扶贫规模。大力扶助弱势群体，进一步推进和完善城乡最低生活保障制度。建立健全住房保障体系，逐步普及住房公积金保障制度。千方百计为民办实事，让更多群众得到实惠。高度重视社会公平，合理调节收入分配。加强劳动保障监察执法，贯彻落实最低工资制度，推进务工人员劳动合同签订和劳动争议仲裁体制改革，维护外来务工人员的合法权益，促进劳动关系和谐稳定。

努力提高社会保障水平。继续实施积极的就业政策，拓宽就业渠道。加快建立统一规范、城乡一体的市、县两级劳动力市场，进一步完善公共就业服务体系。努力扩大就业，力争就业人数和农村劳动力转移人数达到6.75万人和15万人，“十一五”期间从业人员达到175万人，城镇登记失业率控制在4.6%以内。完善各种保险制度，健全社会保险市级统筹机制。扩大社会保险覆盖面，完善农民工医疗保险制度和工伤保险制度。改进养老金管理办法，解决好破产企业和困难企业职工参加基本医疗保险和养老保险问题，建立农村社会保险制度。积极推进企业退休人员社会化管理，加强社会保险基金征缴和监管。

强化社会治安综合治理。加强和改进信访工作，认真对待和合理解决群众反映的问题。加强社会矛盾排查调处工作，积极预防和妥善处理群体性事件。完善突发公共事件应急处置机制，增强应对突发公共事件的能力。开展“五五”普法教育，进一步提高全民法制意识。全面推进“平安梧州”建设，建立健全社会治安防控体系和公共安全防控体系，增强人民群众的安全感。依法严厉打击各种刑事犯罪，坚决遏制“黄赌毒”和非法传销的滋生蔓延。加强治安防控的科技装备建设，提高治安管理水平。加强对流动人口和出租屋的社会化管理。

提高公共安全保障能力。建立健全各种自然灾害和公共安全突发事件监测预警体系和信息报告、应急救援、社会动员机制，增强全民防灾抗灾意识，提高应对处置公共安全、突发事件能力。加强食品、药品安全的监督管理。加快地质灾害整治步伐，全面整治地质灾害，创造一个适宜人居的生活环境。以防范重特大安全事故为重点，加大安全生产综合监管和执法力度，强化安全生产责任制和责任追究制。加大投入，改善安全生产条件，严格安全生产许可制度和安全“三同时”审批制度，促进安全生产形势稳定好转。

八、着力推进“科教兴市”战略实施，加快发展社会各项事业

今后五年要把发展社会事业放到重要位置，努力促进经济建设与社会各项事业全面、协调和可持续发展。

抓好精神文明建设。全面贯彻落实《公民道德建设实施纲要》，深入开展社会主义荣辱观教育活动。围绕提高市民素质和城乡文明程度，扎实开展各项文明创建活动，努力创建自治区第五轮文明城市。加强未成年人思想道德建设，为经济社会发展营造健康向上的文化氛围和稳定和谐的社会环境。

加强科技和人才建设。大力推进工业科技创新，鼓励和引导企业增加对科技的投入。继续抓好向农村选派科技特派员工作，带动农村科技进步。广泛开展科普活动，推广实用技术，提高全民科技文化素质。加强多层次人才队伍建设，落实促进人才发展的各项政策措施。创新人才管理机制，加快人才小高地建设，培养引进高层次、复合型、高技能人才，努力创造人才辈出、人尽其才、才尽其用的新局面。

坚持把教育放在优先发展的战略地位。全面实施素质教育，进一步调整优化中小学布局，合理分配教育资源，促进各类教育协调发展。进一步完善“以县为主”的农村九年义务教育管理体制，继续加大中小学危房改造力度，在全面普及和巩固九年义务教育的基础上，加快普及高中阶段教育。大力发展中、高等职业教育，增加对职业教育的投入，扩大职业教育办学规模。进一步办好梧州学院，提升学院办学质量，充分发挥高等教育在地方经济建设中的作用。加强教师队伍建设，全面推广应用现代教育技术，提高教育教学质量。建立有效的教育资助体系，帮助贫困家庭学生完成学业。积极引导、鼓励和扶持社会力量办学，推进民办学校的发展壮大。

完善公共卫生和医疗服务体系。强化政府职能，加大政府对卫生事业的投入，进一步建立健全突发公共卫生事件应急机制，不断提高疾病预防控制和医疗救治服务能力，防范重大疫情发生和有效控制结核病、艾滋病等重点传染病。深化医疗卫生体制改革，重点加强农村三级卫生服务网络和以社区卫生服务为基础的新型城市社区卫生服务体系建设，进一步完善乡村一级卫生基础设施建设和新型农村合作医疗制度，推进医疗卫生人才培养和妇幼保健工作，努力为广大人民群众提供安全、有效、方便、价廉的公共卫生和基本医疗服务。

积极发展文化体育等社会事业。深化文化体制改革，发展文化产业，重点打造宝石文化、龙母文化、宗教历史文化和名人文化品牌，努力培育一批有自主创新能力、有知名品牌、有自主知识产权的文化企业，大幅度提高文化的整体实力和竞争力。继续实施文化精品战略，提升群众文化活动水平，不断繁荣新闻出版、广播影视、文化艺术。加强对外文化交流，营造健康向上的社会文化环境。加强城乡社区文化体育设施建设，加快市体育中心、青少年活动中心、妇女儿童活动中心、博物馆、图书馆、文化广场等标志性文化设施建设。广泛开展群众性体育运动，推动全民健身运动深入开展，提高竞技体育水平。全面贯彻新修订的《妇女权益保障法》，切实保障妇女、未成年人、残疾人的合法权益。关心老年人身心健康，做好老龄工作。加强全民国防教育，增强国防意识，提高国防动员、民兵预备役和双拥工作水平。做好民族、外事、侨务、对台事务、审计、统计、民政、气象、人防、档案、修志、地震、社会科学、宗教等工作。大力支持各民主党派、工商联以及工会、共青团、妇联等人民团体开展工作，充分发挥他们在构建和谐社会中的重要作用。

加强人口、资源和环境保护。建立健全计划生育工作新机制，稳定低生育水平，减少政策外生育，提高人口素质，统筹解决人口问题，人口自然增长率控制在11‰以内。强化节约意识，以工业节能为重点，大力推广节能节水节地节材，努力建设节约型社会。发展循环经济，积极开发和推广循环利用技术，探索发展循环经济的有效模式。加强生态建设，加强污染治理，健全环境监管体制，提高环境监管能力。

九、着力推进政府职能转变，不断提高依法行政能力

今后五年，政府要加快推进职能转变，不断提高依法行政能力，努力建设一个廉洁、勤政、务实、高效的政府。

加强民主法制建设。坚持自觉接受人大及其常委会的监督和人民政协的民主监督，坚持向人大及其常委会报告工作和向人民政协通报情况的制度。办好人大代表议案、建议、批评和意见以及政协委员提案。坚持科学民主决策，完善重大事项民主协商、决策参与和民主监督制度，提高决策水平。全面实施《依法行政实施纲要》，严格执法、公正执法、文明执法，努力提高政府依法行政水平，推进依法治市进程。

努力建设勤政高效的服务型政府。以转变政府职能为重点，推进行政管理体制改革，切实把政府职能转到为市场主体服务、创造良好发展环境和建立健全公共服务体系上来。全面履行政府职能，提高预防和处置突发公共事件的能力。依法实施行政许可，大力推行首问负责制、限期办结制，进一步提高政府机关的办事效率，全面推行行政执法责任考核考评制度，实行行政过错追究制度，着力提高政府公信力。

认真贯彻执行《公务员法》。进一步规范公务员队伍的教育管理，建设一支政治强、作风实、业务精、廉洁自律的公务员队伍。牢固树立“清简务本，行必责实”的工作作风，努力为基层和群众办好事实事。

深入开展反腐倡廉工作。建立健全教育、制度、监督并重的惩治和预防腐败体系，从源头上预防和治理腐败。继续加强政府投资监管、国有资产监管以及建设工程招投标、经营性土地使用权出让、政府采购和国有产权交易监管。严格执行问责制度、干部轮岗交流制度、任职回避制度、经济责任审计制度等。严肃查处以权谋私、权钱交易等腐败案件，加大治理商业贿赂和预防腐败工作力度，坚决遏制腐败现象发生。

各位代表，站在新的起点上，面对着无尚光荣的使命和更加崇高的目标，我们要更加紧密地团结在以胡锦涛同志为总书记的党中央周围，高举邓小平理论和“三个代表”重要思想伟大旗帜，全面落实科学发展观，在市委的坚强领导下，在市人大的监督和市政协的支持下，紧紧依靠全市各族人民，解放思想，与时俱进，同心同德，开拓创新，为加快建设富裕和谐新梧州而谱写无愧于我们伟大时代的新篇章！
